# A bridge from uncertainty to understanding: The meaning of symptom management digital health technology during cancer treatment

**DOI:** 10.1177/20552076231152163

**Published:** 2023-01-22

**Authors:** Andrew Darley, Barbara Coughlan, Roma Maguire, Lisa McCann, Eileen Furlong

**Affiliations:** 1 School of Medicine, 8797 University College Dublin, Dublin, Ireland; 2 School of Nursing, Midwifery and Health Systems, 8797 University College Dublin, Dublin, Ireland; 3 Digital Health and Wellness Group, Department of Computing and Information Sciences, 3527 University of Strathclyde, Glasgow, UK

**Keywords:** Digital health, cancer, family caregiving, qualitative research, interpretative phenomenology

## Abstract

**Objective:**

Digital health technology is valued as a tool to provide person-centred care and improve health outcomes amongst people with cancer and their family caregivers. Although the evidence to date shows encouraging effectiveness, there is limited knowledge regarding the lived experience and personal meaning of using supportive technology during cancer treatment. The aim of this study was to explore the lived experiences of people with colorectal cancer receiving chemotherapy using digital health symptom management technology and their family caregivers.

**Methods:**

A longitudinal and multi-perspective interpretative phenomenological analytical approach was adopted including three people with newly diagnosed colorectal cancer and four family caregivers.

**Findings:**

Three superordinate themes and related subthemes were identified. The first theme (The 3 Cs of symptom management technology) centred on the continuity of care that participants felt while using the technology. The second theme (Digital health technology as a psychosocial support) offered insights into the psychological benefits using technology incurred as they navigated their cancer diagnosis including sense of control and psychological safety. The final theme (Impact of digital health technology on family caregivers) details the supportive effect the technology had on family caregivers’ role, responsibilities and well-being during the cancer experience.

**Conclusion:**

Digital health technology can act as a bridge from uncertainty to an understanding regarding a cancer diagnosis and its treatment. Digital health technology can support peoples’ understanding of cancer and enhance self-management practices, while being a psychological support in navigating the uncertain and often worrying period of receiving cancer treatment.

## Background

Digital technology and the Internet have aided a reimagining of how healthcare can be delivered.^1^ Digital health technology is valued for its ability to place people at the centre of their care, improve health literacy and support decision-making regarding their condition and its care.^2^ Digital health technology is particularly pertinent for people diagnosed with cancer and their family members. Supporting this population is particularly important considering the shift in cancer care delivery from an in-patient to an outpatient care model, whereby individuals diagnosed with cancer receive their treatment in a cancer centre and return to their home setting. This outpatient care model involves shorter in-patient admissions for people receiving treatment, however, it also means that people with cancer are required to actively monitor and manage potential symptoms and, when necessary, seek support from their cancer care team for symptoms that are significantly burdensome or bothersome.^3^

The outpatient model of cancer care has known challenges, particularly, in relation to reporting treatment-related symptoms amongst people with cancer due to fear of burdening staff, underestimating symptom severity or perception of complaining.^4–6^ More specifically, high symptom burden is also a known risk factor for adverse psychological adjustment and quality of life amongst people with colorectal cancer.^7^ Prip et al.^8^ systematic review found that people with cancer need hope and positivity during cancer treatment which their cancer care team can provide during visits to their cancer care centre, however, this level of engagement with their cancer care team is reduced within the outpatient model of care. The review's findings further underlined how people with cancer valued communication that is delivered in a personal and meaningful way by their cancer care team, including maintaining a compassionate attitude and the ability to convey information effectively.

Internationally, there has been increasing investment and empirical research to identify digital health technologies to support people with cancer in the home setting.^9^ However, fitting digital solutions onto health problems is not an easy task, especially when empirical research studies are often unsuccessful or garner mixed findings with their intended outcomes.^10^ The evidence-base shows how digital health technology can be effective in improving some health outcomes but not others they intend to target amongst people with cancer.^11–13^ The focus of the evidence-base to date has been on the clinical meaning rather than the personal meaning of using digital health technology during cancer treatment. While an understanding of the efficacy and effectiveness are crucial to create and refine technology-based interventions, we also need to understand how the broader effect of digital health and meaning in the lives of its users. A recent review of qualitative evidence in the field^14^ highlighted how digital health interventions can have benefits beyond the intended health outcomes such as feelings of collaboration with their cancer care team and reassurance. Yet, the intent of most qualitative research to date has been to assess the acceptability and usability of digital health technology rather than the conduct of an in-depth exploration of the psychosocial experience or the meaning it had in the personal lives of persons with cancer and family members.

Moreover, to the best of the authors’ knowledge, no study exists which examines what it means to be a family caregiver to a person with cancer using digital health technology. This gap in knowledge is particularly important since family caregivers have a complex role and intimate involvement in the care of people with cancer, in which they have been referred to as ‘the hidden workforce’,^15^(p.136). While family caregivers are believed to be ‘largely consuming the resource’,^16^(p.6), Marzaroti et al.^17^ pointed out that it has been taken as a given that digital health technology is acceptable to this population. Even though some qualitative evidence exists examining the effect of dyadic digital health interventions,^18,19^ no study exists examining the psychosocial experience of using digital health technology for both the person with cancer or those in a caregiver role.

The acceptability, efficacy, and effectiveness of digital health technology are crucial elements to investigate and have shown promising yet inconsistent findings to date.^11–13^ Less is known about the psychosocial experience and meaning of digital health technology to people with cancer and family caregivers. This evident gap needs to be explored and may provide the missing jigsaw piece in order to tailor digital health technology to people's personal values and preferences in their care.

The aim of this study was to explore the lived experiences of people with colorectal cancer receiving chemotherapy using digital health symptom management technology and their family caregivers.

## Methods

A longitudinal, multi-perspective qualitative design using the lens of interpretative phenomenology was employed for this study. Data were collected using one-to-one in-depth interviews with people with colorectal cancer and their family caregivers and analysed using interpretative phenomenological analysis.^20^ Study ethical approval was obtained at University College Dublin and two cancer care centres in Ireland: St James's Hospital and St Vincent's Hospital Group. The research was conducted in accordance with the Consolidated Criteria for Reporting Qualitative Research (COREQ)^21^ and met the specific quality criteria for achieving excellence when using interpretative phenomenological analysis.^22^

### Context of the digital health technology clinical trial

**Figure 1.**
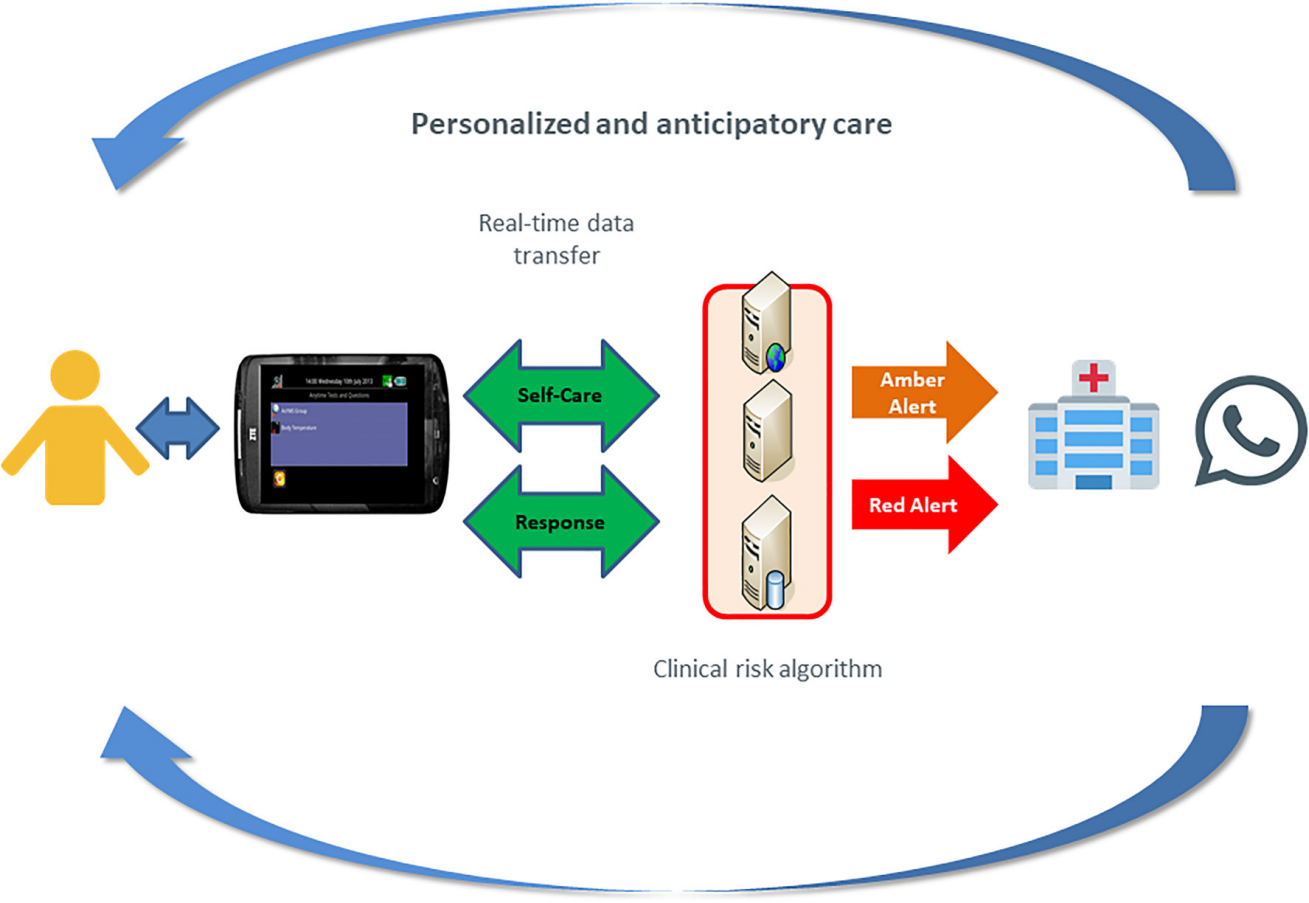
The Advanced Symptom Management System (ASyMS©) care pathway.

The present study was conducted in conjunction with a European, multicentre randomised controlled trial (RCT) examining the effectiveness of electronic symptom management using the advanced symptom management system (ASyMS©) remote technology for patients with cancer.^23^ The primary aim of the RCT, entitled Electronic Symptom Management using the Advanced Symptom Management System Remote Technology (eSMART), was to evaluate the short and long-term impact of the digital health technology on patient-reported outcomes amongst people receiving chemotherapy for breast cancer, colorectal cancer and haematological cancer. Participants were required to report their chemotherapy symptoms daily using the smartphone-based digital health technology. Their information was sent to their cancer care team whose role was to respond by phone or text, depending on the severity of the symptoms. All participants received tailored self-care advice specific to the reported symptoms within the daily questionnaire. The care pathway enabled using ASyMS© is depicted in Figure 1.

### Participants

People with colorectal cancer were recruited from two cancer care centres participating in the eSMART clinical trial in Ireland. As the lead author was also employed as a Researcher/Project Manager in the clinical trial, research nurses acted as gatekeepers in identifying potential participants, assessing their interest in the study and obtaining verbal consent to be contacted by the researcher. The researcher provided interested participants with an information leaflet and consent form detailing the aim of the study and what would be required of them if they chose to participate. All three participants with colorectal cancer who were approached decided to participate in the study. Each participant was asked if they would like to nominate a family or informal caregiver to be contacted about the research. This process was not a requirement for their own participation. Two of the participants with cancer chose to nominate family caregivers in the study.

Following the guidlines for research using IPA^20^ a small homogenous sample was recruited to enable a rich interpretation of the participants’ experience. Guided by the need for cancer-specific digital health research,^24^ a purposive sample was included: adults (18+ years) with newly diagnosed Stages I–III colorectal cancer undergoing active chemotherapy using ASYMS© and nominated adult (18+ years) family caregivers. All participants with colorectal cancer were over the age of 70 years and this was their first time being diagnosed and treated for cancer. Of the two people with cancer who nominated family caregivers, one participant nominated their spouse while the second participant nominated their three daughters to take part. All nominated family caregivers identified as female, ranging from 49–71 years of age. Pseudo-anonymised participant details can be seen in Tables 1 and 2.

**Table 1.** Demographic and clinical details of people with colorectal cancer.

Name	Age	Diagnosed (time since diagnosis when first interviewed)	Employment status	Treatment regime	Colorectal cancer staging	Timepoint of initial interview	Time point of follow-up interview
Stuart	71	November 2017 (5 months)	Retired	Surgery and chemotherapy	Stage III	Cycle 4	Cycle 11
Evelyn	78	January 2018 (4 months)	Retired	Surgery and chemotherapy	Stage III	Cycle 4	Cycle 11
Carl	70	January 2018 (5 months)	Retired	Surgery and chemotherapy	Stage II	Cycle 4	Cycle 11

**Table 2.** Demographic and relationship details of family caregivers.

Name	Relative with colorectal cancer	Age	Relationship to relative with colorectal cancer	Time of initial interview (i.e., relative's chemotherapy cycle)	Time of follow-up interview (i.e., relative's chemotherapy cycle)
Faye	Carl	71	Wife	Cycle 6	Cycle 11
Jane	Evelyn	52	Daughter	Cycle 3	Cycle 11
Caroline	Evelyn	54	Daughter	Cycle 4	Cycle 11
Nadine	Evelyn	49	Daughter	Cycle 5	Cycle 11

### Data collection

Prior to each interview, both participant groups were advised that their participation was voluntary and that they could withdraw at any point without consequence to the care provided or participation in the clinical trial. The researcher emphasised that their interview data would be strictly confidential and would not be shared with their relatives or be identifiable in the data analysis. However, people with colorectal cancer agreed for the researcher to contact their specialist nurse in the cancer care centre if they reported experiencing significant levels of cancer distress or became upset during the interview.

Each participant was interviewed on two occasions by the lead author (AD) between March and August 2018. The first interviews with people with colorectal cancer took place during their fourth cycle of chemotherapy while using ASyMS©, while the follow-up interview was conducted during their penultimate cycle after the device had been returned to the research team. Likewise, initial interviews with family caregivers were conducted while their relative was using the ASyMS© device and their follow-up interview was conducted during their relative's penultimate cycle after the device was returned. Adopting a longitudinal phenomenological approach enabled the researcher to further interpret participants’ experiences, which may not have been possible with an interview at one timepoint.^25,26^ A multi-perspective approach using interpretative phenomenology has been argued to foster a more holistic and congruent understanding of phenomenon as it allows for convergence and triangulation to occur within the researcher's interpretation than a single sample can provide.^25^ By including, both people with cancer and their family caregivers, the researcher sought to understand the meaning of the technology in participants’ personal lives and, more broadly, within the family dynamic.

Semi-structured interviews were conducted with participants, with an interview guide containing questions and prompts regarding their experience of using ASyMS© during their cancer experience. Follow-up interviews followed a similar structure but focused on their experience since returning the device while still receiving chemotherapy. This series of interviews also gave the researcher an opportunity to further explore topics that were discussed in the first interview which they believed were valuable to the interpretation of the phenomena. Interviews were conducted in a private meeting room within their cancer care centre, hotel meeting space, or (in some cases) during their chemotherapy session at the request of participants with cancer due to symptom burden. Two family caregiver interviews were conducted via telephone due to personal circumstances. Interviews lasted between 23–95 min and were digitally recorded and transcribed verbatim by the lead researcher.

### Data analysis

All 14 interviews were analysed according to IPA,^20^ an interpretative method of analysis that enables researchers to access participants’ inner cognitive worlds and give voice to participants’ sense-making of their experience. IPA is idiographic and flexible in nature whereby a researcher can immerse themselves in the participants’ lifeworld through iterative reading and coding which facilitates an interpretation of a phenomenon on a psychological level. The lead author conducted the analysis and all coding and interpretations were reviewed and validated by co-authors (BC, EF) to ensure credibility. The process of analysis involved several key stages as outlined by Smith et al.^20^: (a) immersion in the data by reading and rereading the transcript, (b) exploratory coding including initial descriptive, linguistic and conceptual notes, (c) transforming exploratory codes into emergent themes, (d) searching for connections between the identified themes and defining super-ordinate themes, (e) moving to the next interview and repeating analysis process and (f) looking for connections between the cases and developing super-ordinate themes for the whole sample. Although the experiences of both participant groups were viewed as intrinsically connected, the lead author analysed all data from people with colorectal cancer transcripts first before progressing to analyse the family caregiver transcripts. To ensure rigour and transparency, the lead researcher maintained a reflective journal to document how codes and themes developed over time. The identified themes were iteratively challenged and refined through discussions with members of the author team before the final list was agreed. The research team was also guided by Yardley's guidelines for qualitative psychological research.^27^

### Findings

Three super-ordinate themes and related subthemes were identified: ‘The 3 Cs of symptom management technology, ‘Digital health technology as a psychosocial support’ and ‘Impact of digital health technology on family caregivers’, which will be discussed in detail. The extracts presented in this section have been selected as they were deemed most insightful or powerful in embodying the essence of each theme.

### The 3 CS of symptom management technology

#### Communication

A pivotal benefit that participants discussed was how the technology [ASyMS© device] created an effective communication pathway with their cancer care team:

They [the cancer care team] thank me for my contribution but on occasions they have indicated that a member of a clinical team will be back to me and they have come back to me, they been patient with me and listened to what I have to say, maybe some of the stuff that I said was over-cautious but they came back to me and listened to me and they were able to tell me what to do. [Stuart, Interview 1]

Participants believed that they could rely on the technology as a reciprocal communication pathway with their cancer care team to effectively report their symptoms and receive tailored feedback. This belief fostered a feeling of trust both with the technology and their cancer care team. Once participants with cancer completed the daily questionnaire, their concern or worry regarding the symptom was no longer a private issue and the responsibility of addressing them was shared with their cancer care team. Evelyn encapsulated the benefit of shared accountability while using the technology: ‘If there's a problem, someone will ring you’. Carl discussed how the technology was particularly useful during the interim two-week period between chemotherapy treatment when he does not have face-to-face contact with his clinical team. Carl referred to this period as being ‘in between’, implying how Carl feels disconnected or adrift during this period even though he is receiving care. Carl explains that during this time he feels as though he is ‘back to base’ suggesting how he feels unsupported in his home environment in which he must care for himself. Carl identifies the technology as a facility to express the concerns he experiences during this ‘in between’ period. While participants appreciated the ability to communicate with their clinical team, they further valued how the technology enabled and prompted them to give an accurate account of their physical well-being and symptom experience:

The fact that, if you are, if you’re going to fill it in properly, you’re forced to address what your body is telling you. You forced to say, right okay, take your temperature, that's easy stuff but okay but the level of pins and needles that I’m getting, how much does it bother me? Does it bother me slightly? [Carl, Interview 1]

The way in which Carl notes that he is ‘forced’ to answer the questionnaire with accuracy indicates that he may not have disclosed his symptoms to his cancer care team otherwise. The technology encouraged participants not only to objectively report their symptoms regarding their physical wellbeing, but it enabled them to be accurate about what they are experiencing:

You’re able to speak your mind in the mornings, you know when you’re doing the thing, how you feel and what's after happening to you, you know? [Evelyn, Interview 1]

That's great reassurance to know that I can truthfully tell them what my symptoms are or what my side-effects are after treatment and to know that if it's serious or if they’re concerned, they’ll get come back to me. [Stuart, Interview 1]

Evelyn further appreciated how the questionnaire and self-care information is framed: ‘It's in everyone's language, we’ll say, it's not doctor language or anything. It's simple.’ Evelyn's choice of the term ‘doctor language’ signifies how she perceives that her cancer care team use terminology when discussing her care that she finds inaccessible and difficult to understand. This style of communication appears to have made Evelyn feel intimidated and uncomfortable to ask questions or request that the clinicians clarify the information. The effect of this may have made Evelyn feel less involved or able to participate when discussing or making decisions regarding her care. Evelyn appreciates how the technology asks its users questions regarding symptoms in lay terms that are easy to understand, which in turn, provides participants the opportunity to express the nature and extent of their symptoms. Evelyn's use of the word ‘everyone’ implies how she feels that it is an inclusive medium of providing healthcare information, in comparison to face-to-face interactions which she found to be exclusive and difficult to engage in.

#### Connection

The accessibility and frequency of communication, including the daily questionnaire and follow-up phone call or text message, resulted in a sense of connection for the person with cancer to their care team during chemotherapy. Stuart compared this connection to the cancer care team as ‘like having a nurse in the room with you’. This metaphor highlights how using the device was a personable experience to the extent that he felt a nurse was accompanying him in his everyday environment. Evelyn echoed this sentiment in how she believed that ‘there’s someone that’s looking after you’ while using the device. Carl observed how the technology signified a continual reminder that they were connected to their cancer care team who is taking care of them:

Even though I know there's a phone number there that I can call and get through within anywhere from 15 minutes to half an hour or I leave a message, and somebody will call back, it's a nice to have that [the ASyMS© device], you know? [Carl, Interview 1]

Carl further explained that receiving a call from a clinician would ‘break the monotony of my day’ and this was beneficial in between chemotherapy treatments.

#### Clarity

Acknowledging that all participants with cancer had never experienced the condition previously or its treatment-related symptoms, the technology provided clarity regarding the experience. Participants described how it educated them on the symptom experience as they acknowledged the difficulties of retaining symptom management information provided during the pre-chemotherapy education session:

For me, I think so yeah, because you get the symptoms to look for, the pins and needles and things like that, but there's other stuff in there on the phone that's listed, and they do tell you, but you forget. Advise you if you’ve had diarrhoea, constipation, any problem with your feet, which I’ve had, that sort of thing. [Carl, Interview 1]

It's good, yeah, because you can talk to them and they can tell you like what to do. If it's the sickness they tell you about the different tablets and to try and do such and such in between and that yeah, you know? [Evelyn, Interview 1]

Participants appreciated how the daily questionnaire and self-care advice was specific to colorectal cancer and the symptoms they experienced which made the information transparent to understand:

‘The questions that I’m being asked are fairly defined and they’re fairly limited. They’re defined and limited to the illness I have’. [Stuart, Interview 1]

The technology also encouraged users’ self-awareness regarding their bodies and their surrounding environments. This self-awareness fostered by using the technology was particularly helpful to participants in clarifying their abilities during chemotherapy and adjusting their lifestyles accordingly:

It forces the individual or encourages to be more aware of what's happening in their body. You know I’m aware that if I go out on a cold day without gloves, I’m gonna get pins and needles, so I always carry a pair of gloves in my jacket pocket, even on a warmish day, it's just in case, I know if I put my hand on a fridge, I’m gonna get pins and needles. [Carl, Interview 2]

### Digital health technology as a psychosocial support

#### A tool of routine and control

Participants with cancer discussed how the daily practice of symptom reporting became a part of their normal daily routine, as well as how it encouraged them to establish a routine:

You get the phone, it sets you up into a regime, or encourages you to form a regime of what's happening with your body. And afterwards, when you don’t have the phone, you should continue to do that anyways while you’re on the chemo … it's also helping me think this thing through because you could just blindly accept this is what I’ve got here [Carl, Interview 1]

Well, it's em, it's normal if you like. I get up, I have my breakfast, I have a wash and then I come out and I do the questionnaire straight away. That's my rote in the morning. Get up, have a wash, have my breakfast, then sit down and do the questions. It's no imposition on my time really. I do it very quickly. [Stuart, Interview 1]

It's like a ticking clock with me. I watch between 10 and 11 if I’m doing something else, I’ll wait till after. Sometimes I do it around 10 but I know I have to do it and I just do it. [Evelyn, Interview 1]

This routine and repetition of the daily questionnaire appear to have been an anchor in their lives during treatment, which promoted a sense of structure and comfort, as Carl commented ‘I’m one for (pauses) routine. I like, I find a routine for something, it helps me sort of relax a bit’. All participants approached the completion of the daily questionnaire with dedication, as Stuart stated that ‘he wouldn’t miss one’. Evelyn spoke of her sense of ownership and knowledge that she must complete it as part of her care and referred to it as ‘my thing’. As Carl acknowledges that he is someone who has not been previously concerned about his health, he sees how this daily routine of checking his physical well-being on a consistent basis is important during chemotherapy. The device has been an aid to help Carl ‘think this thing through’ as he acknowledges that he could simply accept the symptoms that he experiences without questioning them. The technology has provided Carl with a ‘tool I can use for my own benefit’ which encompasses a feasible routine that he can integrate into his day whereby he checks his own body, which he has been previously unaccustomed to doing. This indicates that the routine of daily checking his physical health has made Carl more mindful of his body and any changes that may occur during his treatment.

#### Psychological safety and reassurance

A pervading theme that connects the experiences of people with cancer is the reassurance they felt from using the technology. The reassurance they referred to was connected to their feelings of being involved and in control of their care by completing the daily questionnaire, as well as the awareness that they can rely on the technology for efficient care should they require it:

Oh, it has definitely helped me. It has helped me in the sense that it has put my mind at rest. I know that if I had a bad night or I had a bad side-effect that there's somebody at the other end of the phone to advise me. They do, they do ring me up and they do tell me what to do and they do warn me to contact them at any time if I, if I need to do that. And that's huge reassurance. [Stuart, Interview 1]

It was very comforting to get the call. It's knowing that there's somebody there eh calling to see how I am in case I’m not well or don’t really know that I’m not well. [Carl, Interview 1]

While both Stuart and Carl described themselves as ‘pragmatic’, it did not inhibit them from experiencing stress regarding their cancer and well-being which they considered the technology provided them with support. Stuart views the technology as having an active role that allowed and supported him to deal with his worries and concerns about his physical well-being, observing that ‘It's somebody minding my back’. This image refers to Stuart's feeling of going through an experience that he feels threatened by or uncomfortable within which he needs protection. Likewise, Evelyn referred to the device as ‘a stand-by if you’re worried’ and how ‘it's a great feeling of knowing someone was looking after your symptoms each day’. This quote signals how Evelyn felt the technology was readily available when she needed help. Evelyn specifically relates this term to experiencing physical symptoms and how it can guard her from them.

Stuart echoed this perception of how technology is a constant safeguard, especially if he has a question or concern: ‘there’s somebody there straight away’. Stuart explained that his feeling of reassurance using the technology is related to how he can disclose the status of his physical well-being to his cancer care team and that ‘if a response is needed to fix how I feel, I’ll get the response’. This implies how Stuart finds reassurance in knowing that he will only be contacted by his cancer care team if his symptoms are significant; noting that the technology device can help ‘fix’ his symptoms if he is unwell. Conversely, Stuart finds it reassuring in knowing that if he does not receive a response from his cancer care team after completing the daily questionnaire, he understands that ‘what’s happening to me is natural’. Stuart's experience highlights how he became dependent on the reassurance that it gave him, as he stated that ‘It’s like you have Mr Reliable, you have we’ll say, you have a back-up’. Again, Stuart refers to the technology as a personable entity that he has become accustomed in knowing and dealing with which promotes a belief that he can depend on it and feel safe.

#### Managing emotions and cancer distress

While the reassurance participants experienced was a comfort for them, the technology had a further role in assisting participants to manage their cancer distress and navigate their emotions. Stuart reflected on how he would cope without technology during his cancer experience:

I would imagine that I would be very stressed out. I’d imagine that I would be thinking all kinds of dire thoughts. I would imagine that I would be, to a very certain extent, left on my own. I certainly think that this is a very positive step in putting my mind at rest for the reasons I set out in that any query I have or question I have, there's somebody there straight away. It's back-up reassurance and certainly if I didn’t have this option or facility opened to me, I would not be as relaxed or as, or as, or as happy as I am, you know? [Stuart, Interview 1]

Stuart catastrophised what his experience would be like without the device which is evident in how he explains that he would ruminate about his symptoms if he did not have it. Stuart explained that, without the technology, the reason he would be concerned is related to how he would ‘probably feel that I was ill and that my symptoms should be treated’. This statement shows the level of worry that Stuart maintained about his cancer and chemotherapy treatment and that these worries could escalate without reassurance from his cancer care team. Similarly, Evelyn discussed how using the technology ‘takes away’ a lot of her worries about her cancer and encourages her to ‘not think about bad things as much’. While Carl discussed how using the technology gave him confidence in his cancer experience, he noted that ‘not everybody may react the same way’ – inferring how people's response to the technology and psychosocial benefit may be dependent on each individual's personality and their engagement with it.

### Impact of digital health technology on family caregivers

#### Perceived benefit to a person with cancer

Each family caregiver spoke of how they observed the benefits and positive effects of the technology on their relative which subsequently had positive outcomes for the family caregivers. Faye spoke of how she believed that the technology was a positive force in her and Carl's life as it provided person-centred collaborative and compassionate care:

I think that's helped him in that he's being treated like an intelligent human being because I think like people are intelligent, they have their own sphere of where they work and everybody no matter what they work as em, eh, I think that's sort of helped him that this was something in consultation with him rather than something that is being done to him. [Faye, Interview 2]

Evelyn's daughters discussed the benefit of the routine technology imposed and the knowledge that it is adhering to a routine of reporting their mother's symptoms:

It's now part of her routine. She gets up in the morning, she has her breakfast and does it straight after. She's used to charging it up and putting it back, taking the temperature, she's in that routine and its part of her treatment *…* [Nadine, Interview 1]

Jane described how the device was an empowering tool for her mother who lived alone and how she perceived that it reduced Evelyn's reliance on her family during chemotherapy:

I don’t think she's missed a morning doing it because she knows it's a back-up for her because she got calls back a couple of times from some of the stuff she does be saying, which is brilliant for her when she's not there, you know when there's nobody else around with her [Jane, Interview 1].

#### Tool of support in providing support

Family caregivers discussed how the technology was not only an educational and supportive tool for the relative with colorectal cancer but for them also. The technology was a reliable information resource whereby Caroline compared using the technology to a previous experience with her father's cancer treatment in which she states, ‘I think it’s great work we’re doing and as I said coming from the other side where we had no information’. Jane mentioned her appreciation of the ‘little bit’ of information that the technology enabled regarding specific symptoms and its ability to ‘settle’ the unease or uncertainty within the family of how to address them. The technology also aided family caregivers in identifying symptom patterns during chemotherapy:

You actually knew, we had actually got to know over the course of the two weeks, by using the app and that as well, what day's mammy's sickness would kick in and what days would it start to ease off and, you know, sort of, we had routine then, we sort of knew then, well she's going to be sick now from the Friday till possibly the Monday, you know what I mean and that, because we were able to monitor as well, as she was putting it in on the app [Jane, Interview 2].

This awareness of when to expect symptoms to occur enabled Evelyn's daughters to establish a routine regarding their caregiving duties that were structured in accordance with their mother's symptom pattern i.e., periods when she would need more vigilant care or when she would require family caregiver to stay with her. For Faye, her awareness of Carl's symptom pattern meant that she was able to keep her normal routine, including attending the gym and minding her grandchildren, which she explained was good for her mental health and she could keep her lifestyle routine without ‘hovering over him’. Participants encapsulated how the technology provided a reciprocal sense of control to family caregivers when supporting a person with cancer:

It supports us. It supports us. Yes, in supporting her. If the symptoms are going in and we know that she's having these symptoms or whatever she puts into the phone. If they give her a call back to say ‘don’t worry about’ or ‘it's okay, it's fine’ we know then that we can breathe easy and we don’t have to worry about bringing her to the doctor, that it's quite normal, you know. They never told us with daddy that he would have horrendous nausea and that it was going to be quite normal to get that. Whereas with mammy we know that if she has something wrong, they let us know what we need to do about it and then we can act from there. So, it supports us in supporting her. [Caroline, Interview 2]

When you’re sort of very ill like that you can feel very much at the mercy of other people, the experts, the people who know what's happening. And you feel so helpless. Because, even, family around feel so helpless too, that, it gives a little measure of control [Faye, Interview 2]

#### Facilitator of communication with cancer care team and within the family

Family caregivers observed how their relatives' daily symptom report was their way of ensuring that the cancer care team knew the symptoms being experienced and they could receive direct feedback from the family. The assurance felt from this communication style is evident in Jane's remark of how ‘we know that if there is anything that comes from the survey in the mornings, she is going to get a call back on it’. This observation provides an insight into how family caregivers feel involved in the communication, despite not directly using the technology when their relative provides information to their cancer care team regarding the symptom experience. This sense of involvement in their communication is reflected in how all family caregivers encouraged their relatives to be ‘accurate’ when reporting their symptoms. If their relative with cancer did not correctly report their symptoms, family caregivers viewed this as an obstacle to ‘get the knowledge’ to fulfil their role in providing or supporting symptom management.

The technology was also a facilitator of communication within the family. The technology acted as a focal point which enabled family caregivers an opportunity to discuss the cancer experience especially if a relative did not want to discuss their symptoms. Jane observed how engaging with the device enabled conversations about her mother's current symptoms and how she was feeling, despite her fixation on finishing treatment, which may not have happened without it:

You know, because as I said like, giving her support, she doesn’t want to talk about it really and, you know what I mean. She's just like ‘I want to get this finished’, you know, that's just her big plan now, to just get it over and done with. [Jane, Interview 2]

Conversely, Faye explained how she is characteristically a worrier, as she refers to anxiety as part of ‘my nature’ and how she has to keep ‘my worry from people so to not worry them’. Faye discussed her belief that she can get ‘under people’s feet’ when she is anxious; highlighting her perception that her anxiety may burden or weigh on those she discusses them with. Faye disclosed that when Carl was diagnosed, she believed that she would need to actively care and monitor him during his chemotherapy treatment: ‘one of my worries initially was would I have to keep hounding him’. Faye perceived how Carl would normally take a passive role in his care and that she would have to actively check on whether he is well, presenting symptoms or in need of clinical care. Simultaneously, Faye notes that Carl can get frustrated when she checks on him and believes that he would prefer if she did not ask questions about his well-being. The technology presented an alternative way of communicating in their relationship; Faye did not need to ask or check on Carl regarding how he was doing because she knew that the technology was doing it for her. As Faye knew that Carl was interested in the technology and the questionnaire, she knew that he was being monitored on an ongoing basis: ‘I didn’t have to worry so much …’*.*

## Discussion

The aim of the current study was to provide a rich interpretative account of the meaning of symptom management digital health technology to people diagnosed with colorectal cancer and their family caregivers during chemotherapy. The study reached beyond the scope of previous qualitative research which focuses on acceptability and usability^14^ and offers an in-depth understanding, on a psychosocial level, of what it means to use digital health technology during cancer treatment and the interpersonal effects it can have within a family.

Daily symptom reporting using the technology facilitated communication, knowledge-sharing and decision-making between people with cancer, family caregivers and the cancer care team. The use of digital health technology in this study acted as a bridge for participants – guiding them from a place of uncertainty to understanding regarding the cancer diagnosis, its treatment and subsequent bodily changes. Acknowledging that information provided during pre-chemotherapy education can be overwhelming and difficult to process^28^ or obtain,^5,29^ digital health technology was a meaningful method to provide participants the information that is tailored, engaging and instantly available. Though the technology may not have provided new information beyond what is currently provided in cancer care education practice, the style of delivery reinforced information specific to their experienced symptoms, as they occurred, which enabled them to continually build their knowledge. As such, digital health technology has the capacity to educate people with cancer and their family caregivers about the experience of living with cancer in an accessible way that suits their learning needs.

The interpretative phenomenological line of inquiry further enabled an understanding of how participants not only learned how to physically manage their symptoms but also how to psychologically *respond* to their symptoms. Using the technology helped demystify the symptom experience as participants became aware of what treatment-related symptoms are supposed to be like, the type of self-care and/or clinical care necessary. The collaborative process between participants and their cancer care team disentangled and overcame known obstructions in reporting symptoms in an ambulatory care setting such as fear of burdening staff, underestimating symptom severity, or reluctance to complain.^5,6^

A central principle of this equal partnership, facilitated by the technology, between families and their cancer care team was the lack of healthcare terminology, which is a known obstruction in cancer care.^30^ Consequently, both people with cancer and their family caregivers felt included in the care process, or rather, they valued how they can collaborate with their healthcare team in helping them. The daily communication resulted in connectedness with their cancer care team which sanctioned their ability to seek help and feel secure in their home setting between chemotherapy treatment sessions, which can be isolating and difficult.^31^

A unique finding of this research pertains to how family caregivers’ experience was impacted by their relative's use of digital health technology. Although they did not personally use the technology, family caregivers experienced reassurance in witnessing their relatives being monitored daily, receiving effective care and engaging in self-management activities. The digital health technology helped family caregivers identify chemotherapy symptom patterns and improved their health literacy. This benefit, in turn, enabled them to prepare and organise the appropriate level of care when they felt their relative would need it. Family caregivers’ increased understanding and hands-on involvement subsequently reduced their worries about their relative and their own uncertainty regarding the need for clinical treatment or ability to manage at home, which are key sources of distress.^16^ Family caregivers’ sense of inclusion in this way is particularly pertinent in light of Leppla and colleagues’^32^ findings regarding how people with cancer wanted their families to be more involved in their care and informed about their condition. Though the digital health technology in the current study was not designed to be directly used by family caregivers, findings suggest that it is possible for family caregivers’ unmet needs to be met through their relative's use of supportive symptom management technology as it alleviated their perceived responsibilities of monitoring health status and decision-making for their relative.

While previous studies have documented some aspects of the lived experience of using digital health technology such as feeling listened to and reassured,^33–35^ these studies did not contain in-depth focus on the psychological processes that underlie these benefits and how technology can affect users’ lives and relationships. The daily requirement of completing the symptom questionnaire within the eSMART clinical trial became an embedded routine in their day, which is regarded as a cornerstone of chronic condition management.^36,37^ Consequently, this embedded daily routine of completing the survey and engagement with their cancer care team promoted people with cancer's health locus of control i.e., their beliefs regarding external or internal control that determines their health.^38^ Similar to how Kretchy and colleagues^39^ found that people with hypertension who had an internal locus of control were more likely to engage in coping strategies focused on solving problems, people with cancer's use of digital health technology fostered their locus of control through their perception of taking preventative measures in becoming unwell. As such, digital health technology can impact peoples’ perception of control regarding their cancer, which can positively frame their cognitive experience and their engagement in self-care behaviours.

Similarly, digital health technology had an impact on family caregivers’ locus of control as it provided them with a sense of control in terms of their perceived responsibility of encouraging and ensuring its daily completion. Acknowledging that monitoring the well-being and treatment-related symptoms of relatives with cancer can be distressing and time-consuming,^40,41^ family caregivers expressed how their relatives’ use of digital health technology enabled them to maintain their own independence and continue with their everyday tasks and hobbies. Family caregivers’ awareness that their relative was being actively monitored by their cancer care team meant that this burden was alleviated and facilitated their autonomy during chemotherapy treatment, as Wang and colleagues^42^ highlighted to be pivotal for family caregivers’ well-being. Additionally, our findings show that family caregivers were able to maintain their familial identity as wife or daughter to their relatives and not fully assume the role of their carer.

Considering the known challenges to communication within families during the cancer experience, such as protecting each other^43^ and difficulties discussing worries,^44,45^ this study indicates how digital health technology can be an external focal point or stimulus for people with cancer and their relatives as a way to discuss and ask questions about their well-being, which they may not feel able to do otherwise. Conversely, in cases where people with cancer did not openly discuss their cancer experience with their family members, the technology assured their relatives and did not need to initiate conversation as they understood their well-being was being actively monitored. Digital health technology can play a mediating role in facilitating communication within the family dynamic about the cancer experience, in which individuals may or may not want to discuss their experience.

### Implications for clinical practice and future research

Several implications for cancer care and potential research areas exist considering the current study findings. Chemotherapy education is a cornerstone of cancer treatment to equip people with cancer and their family caregivers with knowledge regarding their treatment plan and symptom management. While this educational process is traditionally provided before commencing treatment, research has shown that information is often not retained due to feelings of being overwhelmed,^28^ and often people do not report the symptoms they experience^6^ which may prevent learning how to manage them. Current findings support the process of people with cancer being educated through regular, personalised information that they can access in their own time and in their home setting. Our findings show that using this method also involves and empowers family caregivers in the care process, which people with cancer previously expressed.^32^ Moreover, current findings support a reconsidering of the timepoints in which chemotherapy education is delivered as the study suggests that individuals may benefit from routine follow-up education sessions with their cancer care team during chemotherapy, providing a space to ask questions and reinforce previously received information, which is supported by wider literature regarding the memory loss and concentration difficulties related to chemotherapy.^46,47^

The psychosocial benefits experienced by participants in this study suggest that there may be potential for digital health technology to help assess and monitor psychological symptoms in the home setting. Though the eSMART clinical trial did include health outcome measures to assess anxiety and depression, this information was collected to facilitate comparison with control group participants and was not intended to inform clinical treatment. Previous studies on digital health technology which attempted to address physical and psychosocial symptoms have shown mixed and inconsistent findings,^11,48^ this research suggests that the process of assessing physical well-being daily normalises their symptoms and alleviates the difficulty in expressing them to their cancer care team. This observation echoes Loth and colleagues’ findings^49^ which concluded that electronic self-reports regarding psychosocial needs were an appropriate method to establish psycho-oncology referrals and reduce barriers to people's desire to have psychological treatment. Nevertheless, where digital health technology is being tested or implemented within cancer care practice for the management of psychosocial issues, a key consideration is how the clinical setting must have the appropriate staff and skills to appropriately address these experiences. If digital health is to be used to monitor and treat psychological well-being, efficient psycho-oncology services must be available at cancer care centres must be available to support this care pathway.

The coronavirus disease 2019 (COVID-19) global pandemic posed a critical risk to people with cancer in terms of accessing essential cancer care services as individuals were advised not to visit healthcare settings due to the risk of infection^50^ and health systems were forced to reallocate resources for greater acute needs.^51^ The value of digital health technology was amplified during this time in terms of its role in limiting virus spread and protecting people with chronic health conditions by providing convenient access to necessary healthcare services using remote technologies. Using digital health, people with cancer and their families can interact with their cancer care team without the infection risk involved in visiting their cancer care centre or waiting rooms.^52,53^ While current findings show that digital health can support psychosocial well-being and facilitate a sense of connection to their cancer care team during chemotherapy treatment, further work is needed regarding the lived experience of the long-term use of digital health technology as a means of accessing cancer care to ascertain its appropriateness and sustainability.

Though this study prompts further research to evaluate the lived experience of using digital health, this may not be enough to effect meaningful change in this field. Ward et al.^54^ observed how interventions to improve healthcare with proven effectiveness often fail to translate into meaningful care for people with health conditions due to a lack of sustainability. To improve the quality and effectiveness of services, increasing importance has been placed on involving and collaborating with people who use healthcare services.^55–57^ Given that some studies have shown how digital health technology is ineffective or lead to inconsistent results regarding their intended outcomes,^11,12^ the current findings suggest that such outcomes may be because the technology did not align with participants’ experiences or values. The driver of digital health implementation has focused on its meaning in people's clinical care rather than its meaning in people's lives. Steele Gray^58^ recently reasoned that finding psychological meaningful digital health technologies may be a key approach in establishing effective care pathways using such technology. The author argues that there is great value in studying meaningfulness in eHealth as when technology aligns with a person's individual beliefs it is more likely to be used. As such, a value-driven co-design approach^59^ to digital health technology, involving people with cancer, family caregivers, cancer care professionals, technology developers and other key stakeholders, may be a beneficial method to ensure the effectiveness and sustainability of digital health technology in the future. A co-design approach in future studies which would include people with cancer and their family caregivers’ at the design stage may also result in providing additional resources that are user informed rather than assuming and prescribing what is believed to be useful.

### Study limitations

Some limitations must be acknowledged in reading the current findings. While the findings speak of experiences of families during cancer treatment and met the required sample size for IPA,^20^ one participant with cancer chose not to nominate a family caregiver. Though their inclusion may have potentially garnered a richer understanding, the exclusion of their family caregiver reflects the participant's desire to be independent in their care. Additionally, while the sample contained both male and female people with cancer, they were all over the age of 70. The inclusion of participants in a younger age demographic may have resulted in further perspectives regarding the meaning of technology in their lives.

## Conclusion

This study offers a rich, insightful, experiential account of how digital health technology can be a supportive tool in the psychosocial response to cancer and its treatment. If digital health technology is to be widely used in healthcare systems, it is important not only to understand its usability and effectiveness, but also to gain a richer understanding of its psychosocial implications and meaning in the lives of those who use it. While this study presents evidence regarding the lived experience of people with colorectal cancer using digital health technology during the initial sessions of chemotherapy and their family caregivers, further evidence is needed regarding the lived experience of the long-term use of digital health technology as a means of accessing cancer care to ascertain its appropriateness and sustainability.
